# On the Change in Hydrogen Diffusion and Trapping Behaviour of Pearlitic Rail Steel at Different Stages of Production

**DOI:** 10.3390/ma16175780

**Published:** 2023-08-23

**Authors:** Matthias Eichinger, Bernd Loder, Michael Tkadletz, Holger Schnideritsch, Gerald Klösch, Gregor Mori

**Affiliations:** 1 General and Analytical Chemistry, Montanuniversitaet Leoben, Franz Josef-Strasse 18, 8700 Leoben, Austria; 2 Functional Materials and Materials Systems, Montanuniversitaet Leoben, Franz Josef-Strasse 18, 8700 Leoben, Austria; 3 Voestalpine Stahl Donawitz GmbH, Kerpelystraße 199, 8700 Leoben, Austria

**Keywords:** pearlitic rail steel, hydrogen diffusion, hydrogen trapping, electrochemical permeation, thermal desorption spectroscopy

## Abstract

To avoid hydrogen flaking in rail production, it is of crucial importance to understand the differences in hydrogen diffusion and trapping between different production steps. Therefore, as-cast unfinished material was compared with two finished rails, hot-rolled and head-hardened, using electron backscattered diffraction (EBSD), electrochemical permeation, and thermal desorption spectroscopy (TDS). A significant increase in dislocation density was in the head-hardened rail compared with the other material states. This leads to an effective hydrogen diffusion coefficient of 5.8 × 10^−7^ cm^2^/s which is lower by a factor of four than the diffusion coefficients examined in the other states. Thermal desorption spectroscopy analyses show a clear difference between unfinished and finished rail materials. While a peak in activation energy between 32 and 38 kJ/mol is present at all states, only as-cast unfinished material shows a second peak with an activation energy of 47 kJ/mol, which is related to microvoids. The results show that in the investigated material, the effect of increasing dislocation density has a stronger influence on the effective diffusion coefficient than the presence of a second active trapping site.

## 1. Introduction

The international movement of goods is constantly increasing as globalization progresses. Trains are a cost-effective way of transporting large quantities of goods over land. To construct new and maintain existing railway tracks, more than 10 million tons of railway steel are produced per year.

Rail steel classically has a pearlitic microstructure, although rail steel with a bainitic structure is also produced on the basis of modern alloy concepts and heat treatments [1,2,3]. It is known that the applied steels are prone to hydrogen embrittlement (HE), especially to hydrogen flaking [4,5]. This phenomenon describes hydrogen-based cracks which appear in the material hours or even days after production. These cracks lead to a drastic decrease in the material’s mechanical properties and fatigue strength [6,7,8]. Hydrogen uptake during the production process, in combination with internal stresses (resulting from phase transformations, temperature differences, or deformation) and a prone microstructure, has been identified as the main reason for hydrogen flaking [7]. To reduce the hydrogen content, a vacuum treatment is mandatory for most rail steel before casting. The only production unit between vacuum degassing and continuous casting and, therefore, the place where most hydrogen is taken up by the steel bath is the tundish, where hydrogen can be provided due to the humidity of refractory lining or casting powder [5,9]. During solidification near eutectoid, melts traverse several phase transformations with different hydrogen solubilities. The liquid metal has the highest solubility for hydrogen, followed by the face-centered cubic austenitic lattice structure, which can hold significantly higher amounts of hydrogen compared with a body-centered cubic ferritic one [10]. As the solidification of the bloom progresses from the outside to the inside during continuous casting, hydrogen, which can no longer be dissolved in the solidified outer layers, is absorbed by the liquid phase in the center of the bloom. This results in an enrichment of hydrogen from the outside to the inside of the bloom. Over the course of the rail rolling process, the bloom is reshaped in such a way that the former center of the bloom (high hydrogen content) is located at the transition from the rail neck to the rail head and, thus, at the area with the narrowest cross-section. Moreover, this section is subjected to the fastest cooling rates during heat treatment. Hydrogen diffuses as a function of temperature, time, and microstructure in the matrix of the material and accumulates at trapping sides such as dislocations, grain boundaries, phase boundaries, vacancies, microvoids, or non-metallic inclusions. If a sufficiently high enrichment of hydrogen atoms takes place at such trapping sides, the lattice cannot dissolve the hydrogen anymore, and it precipitates as molecular hydrogen. This process is linked to a high volume expansion resulting in high internal stresses that can lead to crack formation [11]. 

Although the problem of hydrogen flaking has been present in the manufacturing of rail steel for a long time, detailed studies about hydrogen diffusion and trapping behavior at different steps in the production of rail steel are rare in the literature. Deep knowledge of these processes can significantly improve the production of rail steel, especially regarding the heat treatments applied to reduce the material’s hydrogen content. In this context, this study aims to provide a detailed analysis of hydrogen interactions in eutectoid rail steels with a special focus on the changes in hydrogen diffusion and trapping concerning different production steps and heat treatments. To achieve this objective, three material conditions corresponding to different production steps (pre-cast material, hot-rolled rail, and head-hardened rail) are investigated using a combination of high-resolution electron microscopy, electrochemical permeation, and thermal desorption spectroscopy (TDS).

## 2. Materials and Methods

### 2.1. Materials

**Figure 1:**
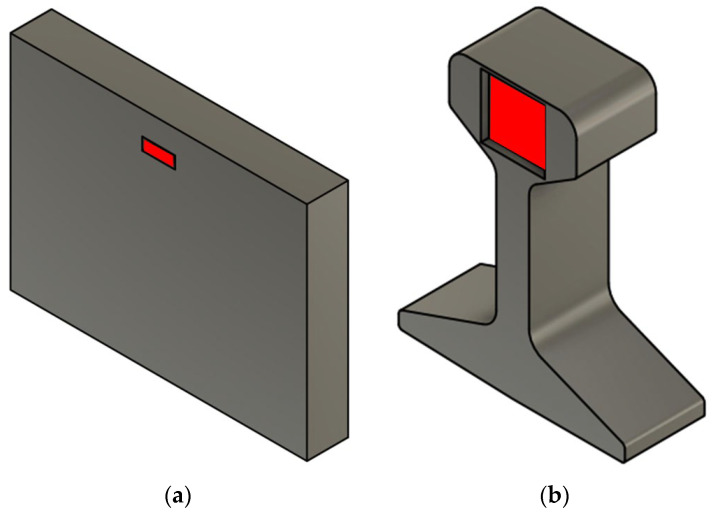
Positions of sample machining (red) of (**a**) As-cast and (**b**) HR and HT material condition.

The investigated material is a eutectoid low alloyed steel with a chemical composition of 0.79 wt.% C, 0.30 wt.% Si, 1.06 wt.% Mn, 0.016 wt.% P, 0.014 wt.% S, 0.051 wt.% Cr and 0.0005 wt.% Ti. In secondary metallurgy, the steel was degassed to reduce the dissolved hydrogen content below production limits prior to tundish treatment and continuous casting. To avoid hydrogen flaking of the finished rails, blooms were stored in effusion boxes to reduce their hydrogen content. Thereafter, the material was heated up to 1000 °C and rolled into rails. To analyze the diffusion and trapping behavior of the as-cast (As-cast) bloom, specimens were taken at 75% of the bloom’s height. In the course of this study, the finished rail materials were examined in hot-rolled (HR) as well as in heat-treated (HT) conditions, i.e., head-hardened conditions. Therefore, samples were manufactured out of the rail head. Figure 1 gives the exact sampling positions (highlighted in red) for all states. The abbreviations in brackets indicate the further designation of the different material conditions in the text.

### 2.2. Materials Characterization

The microstructure of all samples was characterized across scales using a light optical microscope (LOM) (Olympus, Tokyo, Japan) and a scanning electron microscope equipped with an electron backscattered diffraction (EBSD) detector. The investigations using the LOM were conducted on an Olympus AX70. The investigated specimens were hot embedded in epoxy resin and ground stepwise with SiC abrasive paper of grits 120, 240, 500, and 1000. Thereafter, samples were polished with 1 µm diamond paste and etched using 3% nital solution for 10 (As-cast, HR) to 30 (HT) seconds. Additionally, a cross-section of the As-cast conditions bloom was prepared by etching it with a mixture of 50% HCl and 50% H_2_O at 60 °C. Furthermore, the material’s hardness (HV1) was determined using a fm-300 (Future Tech, Kanagawa, Japan) hardness testing device.

In order to avoid mechanical deformations caused by the sample preparation, the cross sections for the EBSD investigations were produced using an IonSlicer (Hitachi IM4000+, Tokyo, Japan). For sample preparation, an excitation voltage of 6 kV and an oscillation of 15°/6 per minute were applied for 60 min. EBSD analyses were carried out using a GeminiSEM 450 (Zeiss, Jena, Germany) field emission SEM coupled with an Symmetry EBSD detector (Oxford Instruments, Abingdon, UK). A step size of 200 nm was chosen for the investigations. Imaging was conducted using the software AZtec 5.1 as well as AZtecCrystal 2.1.

The dislocation densities of all material states were determined using X-ray diffraction (XRD) with a Bruker D8 Advance Davinci (Bruker, Billerica, MA, USA) diffractometer, which was equipped with a Cu-K_α_-X-ray tube. The measuring procedure and the selected parameter settings are described in detail in [12]. To evaluate data regarding the dislocation density, whole powder pattern modeling was conducted using a Topas (Bruker, Billerica, MA, USA) macro based on the theory of Krivoglaz and Wilkins [13,14,15]. 

### 2.3. Electrochemical Permeation Testing

For the permeation measurements, square-shaped samples with a cross-section of 40 × 40 mm and a thickness of 1.2 mm were manufactured. The thickness of these specimens was further reduced upon grinding with SiC abrasive paper with grit 1000 to a final thickness of 1.000 ± 0.005 mm. A 100 nm thick palladium layer was applied on one side of the sample using physical vapor deposition. The specimen was inserted into a double cell, according to Devanathan and Stachurski [16], in such a way that the palladinated side was facing the cell’s oxidation side. The electrolytes used for the permeation experiment were 0.1 M NaOH on the oxidation side and 3.5% NaCl solution with the addition of 1 g/L SC(NH_2_)_2_ on the charging side, respectively. To ensure a minimum amount of dissolved oxygen, both electrolytes were continuously purged with Ar (99.999%). The experiments were conducted at 25 °C, and a charging current of 1 mA/cm^2^ was applied. The potential on the oxidation side was kept constant at +540 mV against a standard hydrogen electrode, equal to +300 mV against a standard calomel electrode. Two charging cycles were performed on each material condition. These measured data were evaluated using the standardized time-lag method according to Equation (1) [17]:

(1) $$D_{eff}=\frac{L^{2}}{6\cdot t_{lag}}$$

where *D_eff_* is the effective diffusion coefficient of hydrogen, *L* is the sample thickness, and *t_lag_* is the time between applying the charging current and reaching 63% of the oxidation currents plateau value.

### 2.4. Thermal Desorption Spectroscopy

TDS spectra of all materials were recorded using a Bruker Galileo G8 (Bruker, Billerica, MA, USA) coupled with an IR07 infrared furnace and an IPI quadrupole mass spectrometer to determine the active traps in the sample materials. For this purpose, specimens with a geometry of 30 × 6 × 1.2 mm were manufactured and electrolytically charged with hydrogen. These specimens acted as the cathode and were placed in the center of a platinum mesh electrode which served as the anode to secure homogeneous hydrogen charging. Charging was conducted at 10 mA/cm^2^ for one hour at 25 °C, where 0.5 M H_2_SO_4_ with the addition of 1 g/L SC(NH_2_)_2_ served as electrolyte. After charging, the specimens were flushed with acetone and immediately cooled in liquid nitrogen. Prior to recording the TDS spectra, the specimens were held for ten minutes at room temperature (25 °C) in air to remove hydrogen adsorbed onto the surface. This was performed to prevent interference with the measured signal originating from the hydrogen effusing from the traps. The system was calibrated with a calibration gas consisting of nitrogen and 5.05% H_2_. For the measurements, N_2_ (99.999%) was used as the carrier gas. The recorded TDS spectra were conducted under the application of four different heating rates (200, 400, 800, and 1200 K/h), during which the specimens were heated from 25 to 450 °C. To ensure no deep traps resulting in high-temperature peaks were missed, selected tests were heated up to 900 °C. Peak deconvolution was performed using the PeakFit v4.12 software, applying a Gaussian distribution for all single peaks. The activation energies for the different peaks of TDS spectra were calculated according to Kissinger’s approach after Equation (2) [18,19]:

(2) $$\frac{d\left(\ln\frac{\phi }{T_{p}^{2}}\right)}{d\left(\frac{1}{T_{p}}\right)}=-\frac{E_{A}}{R}$$

where *ϕ* is the heating rate, *T_p_* is the temperature of the peak center from the TDS spectrum, *E_A_* is the activation energy for the desorption of hydrogen from the specific trap, and *R* is the universal gas constant.

## 3. Results

### 3.1. Materials Characterization

**Figure 2:**
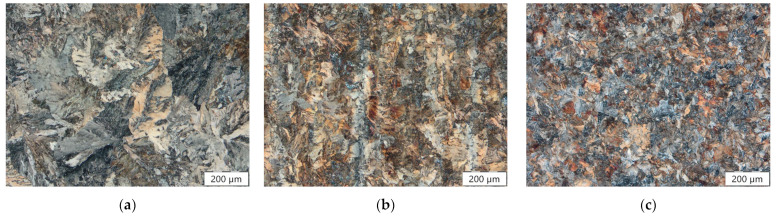
Microstructure of the (**a**) As-cast, (**b**) HR, and (**c**) HT states observed using LOM.

**Figure 3:**
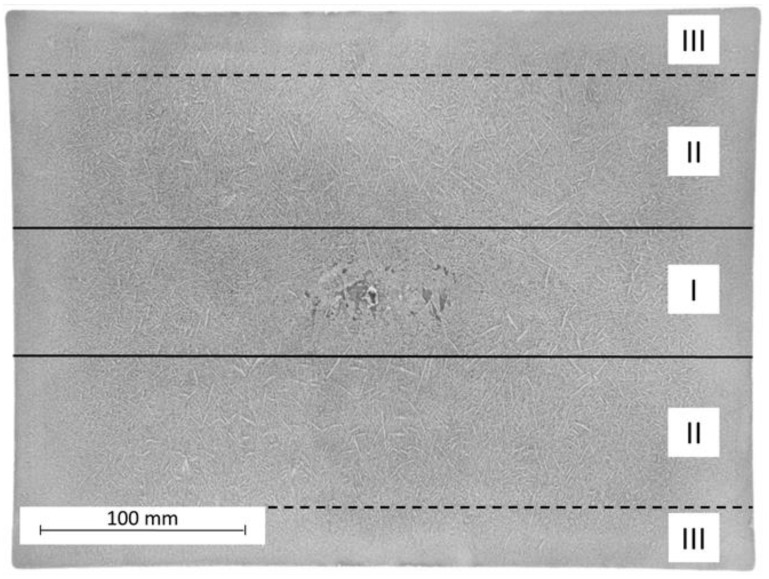
Cross-section of the As-cast bloom with indications of different regimes (I to III) regarding the presence of blowholes, pores, and microvoids. Regime I represents the blooms center with large sized blowholes and pores. In regime II minor pores, blowholes and microvoids are present. Regime III is free of blowholes, pores and microvoids.

Figure 2 gives the microstructure of As-cast (a), HR (b), and HT (c) conditions analyzed using LOM at a 100-fold magnification. Although a purely pearlitic microstructure is visible for all conditions, the appearance of the pearlite colonies changes significantly depending on the material’s condition. The As-cast state has the coarsest microstructure of all the materials examined; its pearlite colonies are oriented toward the dendritic solidification and have a diameter of several hundred micrometers. A decrease in the average pearlite colony diameter compared to the As-cast condition is observed for the HR (around 100 µm) and the HT (<100 µm) conditions. Furthermore, the pearlite colonies in the HR as well as in the HT state are oriented randomly and, therefore, independent from the material’s prior solidification direction. In Figure 3, the bloom’s cross-section is provided. As indicated by the full and dashed lines, the cross-section can be divided into three regimes marked as I to III. Regime I represents the bloom center where big blowholes and pores are present. The size and density of these defects decrease with increasing distance from the bloom center so that in Regime II, minor pores, blowholes, and microvoids were observed. Regime III, closest to the surface, is free of pores and blowholes.

The hardness measurements show a significant increase for the HT condition (364 ± 8 HV) compared with the As-cast (278 ± 3 HV) and the HR (285 ± 7 HV) states.

**Figure 4:**
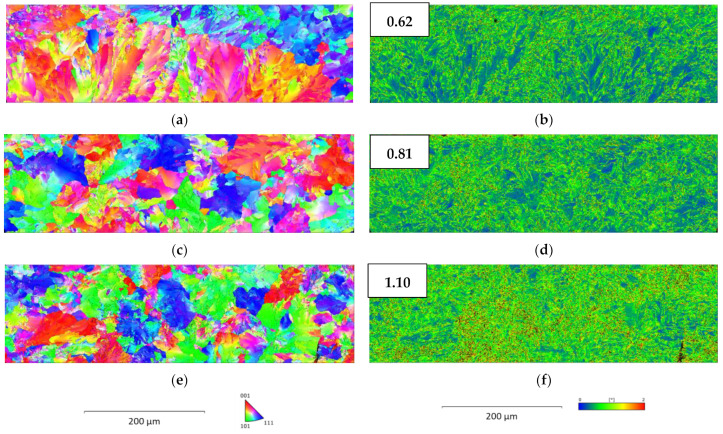
IPF maps in the x-direction of (**a**) As-cast, (**c**) HR, (**e**) HT state, and KAM factor map with an indication of the average KAM factors of (**b**) As-cast, (**d**) HR and (**f**) HT condition.

The results of the EBSD investigations are given in Figure 4 in the form of inverse pole figure (IPF) maps in the x-direction ((a), (c), and (e)) and Kernel average misorientation (KAM) maps (b), (d) and (f)) for all samples. Additionally, the average KAM factors are given in Figure 4a–c. The crystallographic orientation distribution of the As-cast material (Figure 4a) shows a relevant orientation texture over large parts of its cross-section. For HR (c), the IPF maps show a significantly decreased fraction of textured microstructure compared to the As-cast state. The finest grain size with a completely random distribution of grain orientation is shown for the HT (e) material. The amount of zones with low KAM factor (blue) decreases from the As-cast (b) to HR (d) condition and further nearly disappear in the HT (f) state. In the HT material, there is a significantly higher proportion of areas with a high KAM factor (red) compared with other conditions. This results in average KAM factors of 0.66 for the As-cast, 0.74 for HR, and 1.10 for the HT conditions. Figure 4b shows that the zones with the lowest KAM factors are located in sample areas of highly oriented texture. The black areas in Figure 4 are due to insufficient resolution in these sample areas. Apart from IPF and KAM, the fractions of body-centered cubic (bcc) and face-centered cubic (fcc) lattice structures were determined using EBSD, resulting in 100% bcc and 0% fcc microstructures for all investigated materials. The fraction of low-angle grain boundaries (LAGB) and high-angle grain boundaries (HAGB) are comparable for the HR and HT conditions and resulted in about 80% of LAGB (with an angle ≤ 15°) and 20% HAGB (with an angle ≥ 15°) respectively. In comparison, the As-cast condition has a lower proportion of HAGB at 11.8%.

XRD measurements prove a significant increase in the dislocation density from 2.1 × 10^14^ m^−2^ for the As-cast material to 3.9 × 10^14^ m^−2^ for the HR and 9.2 × 10^14^ m^−2^ for the HT conditions.

Summing up, one can conclude that significant differences between the microstructures of the As-cast, HR, and HT conditions are present. The processes in rail production lead to an increasing refinement of the microstructure from the As-cast to the HR and HT state. This simultaneously leads to a reduction in texture and an increase in hardness as well as the KAM factor and the dislocation density. In addition, the annealing and forming treatments close blowholes, pores, and microvoids. A qualitative summary and comparison of the microstructural features of all investigated material states is provided in Table 1.

### 3.2. Electrochemical Permeation Measurement

**Figure 5:**
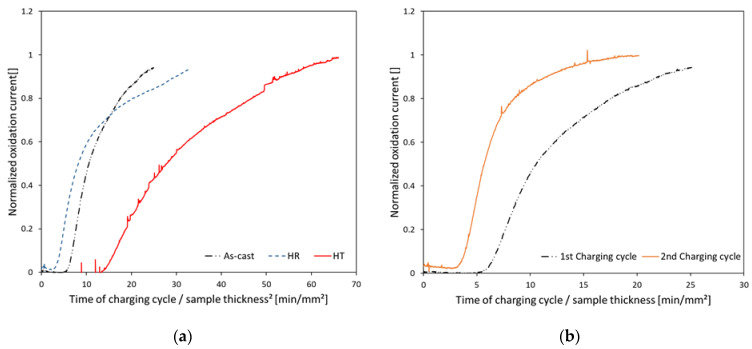
(**a**) First charging cycles permeation transients of all material conditions, (**b**) Comparison of permeation transients of the As-cast materials first and second charging cycle.

To determine the influence of hot rolling and different heat treatments on hydrogen diffusion, electrochemical permeation tests were conducted on all material conditions. Figure 5a shows the permeation transients of all states during the first charging cycle. It is shown that the permeation transients of the As-cast and HR material do not differ significantly as the current rises as a function of time. There are minor differences in the steepness of corresponding transients for these two material states. In comparison, the HT state shows different behavior in terms of both criteria. The time lag was two times higher compared with the As-cast and HR conditions. Apart from this, the slope of the HT permeation transient is less steep compared with the ones from the As-cast and the HR state. The effective diffusion coefficients of the first (*D_eff_*_1_) and second (*D_eff_*_2_) charging cycles obtained from electrochemical permeation experiments are shown in Table 2 for all states. It can be seen that *D_eff_*_1_ is equal for As-cast and HR. Compared with that, the HT material provides a four times lower effective diffusion coefficient in the first charging cycle. The effective diffusion coefficient of the As-cast condition increases by nearly a factor of two between the first and second charging cycles. To illustrate this behavior, Figure 5b gives the permeation transients for the first and second charging cycles of the As-cast state. It is shown that the time lag until an increase in oxidation current occurs significantly decreases from the first to the second charging cycle. In terms of HR, just a slight increase in the effective diffusion coefficient was measured for its second charging cycle, and the effective diffusion coefficient of the HT state was unaffected by the number of applied charging cycles. The permeation transients of HR and HT do not show a change between the first and second charging cycles comparable to the one of the As-cast state.

### 3.3. Thermal Desorption Spectroscopy

**Figure 6:**
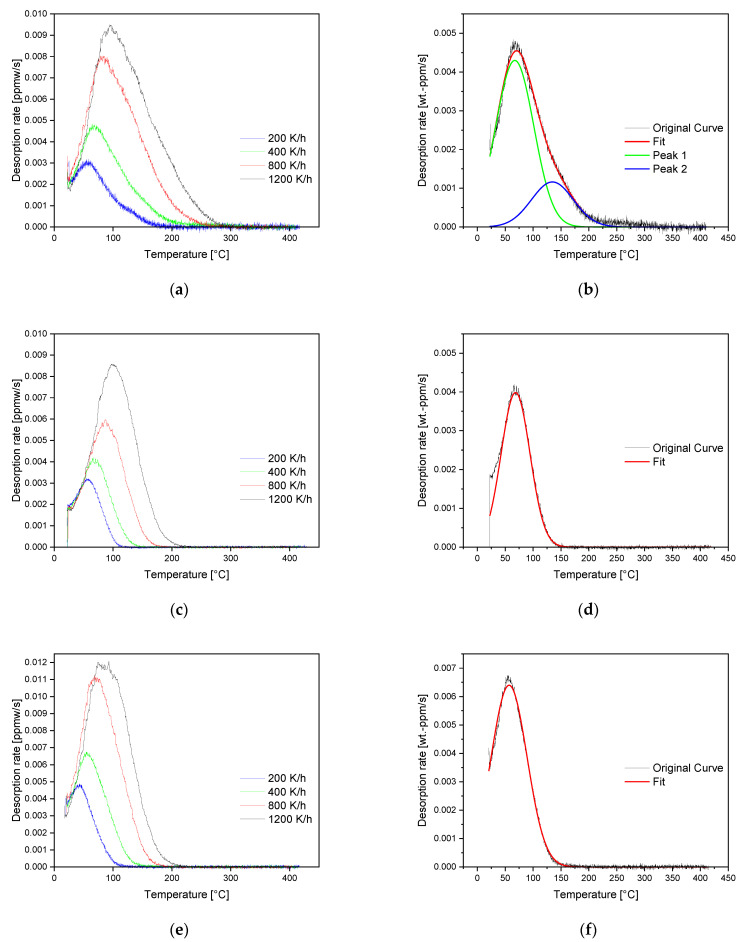
Desorption rates according to the applied heating rates for (**a**) As-cast, (**c**) HR, (**e**) HT and peak deconvolution of desorption spectra obtained at 400 K/h for (**b**) As-cast, (**d**) HR, and (**f**) HT condition.

**Figure 7:**
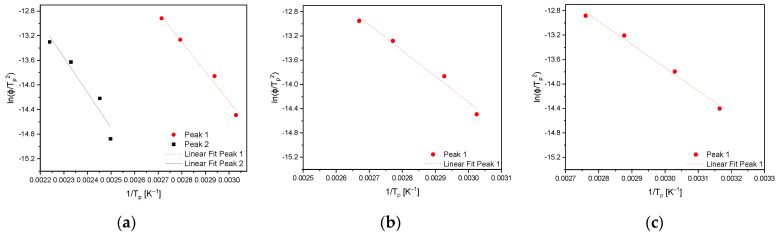
Plots of ln(*ϕ*/*T_p_*^2^) as a function of 1/*T_p_* for (**a**) As-cast, (**b**) HR, and (**c**) HT material.

Figure 6 shows the desorption spectra of the As-cast (a), the HR (c), and the HT (e) material obtained by applying all four heating rates and the corresponding peak deconvolutions at a heating rate of 400 K/h for As-cast (b), HR (d) and HT (f) condition. The obtained desorption rates increase, with the heating rate reaching its maximum values at a heating rate of 1200 K/h. For the As-cast and HR states, they are close to each other and in the range of 0.0085 to 0.0095 wt.-ppm/s. The HT condition, compared with the other material conditions, has a slightly increased maximum desorption rate of 0.012 wt.-ppm/s. Considering the peak deconvolution, it can be seen that the measured sum curve of the As-cast condition (b) can be fitted by two peaks and reaches baseline at 250 °C. Therefore, 250 °C can be considered the temperature where all the hydrogen absorbed during charging has effused from the specimen. In contradiction to the As-cast state, the spectra of HR (d) and HT (f) consist of one single peak and reach their baselines at about 150 °C. Based on the spectra of peak deconvolution, it can be seen that the maxima of the As-cast material’s first peak and the peaks from HR as well as HT all occur in a close temperature range between 60 and 70 °C. The peak maximum of the As-cast material’s second peak is shifted towards higher temperatures and located at 130 °C. The plots of ln(*ϕ*/*T_p_*^2^) as a function of 1/*T_p_* are given in Figure 7, where *ϕ* represents the heating rate, and *T_p_* is the temperature at which the peak maximum occurs. The plots depicted in Figure 7 were used for the calculation of the activation energies corresponding to the peaks shown in Figure 6. The slope’s steepness corresponds to the activation energy of the specific trapping site. The peak activation energies gained by the application of Kissinger’s approach are summarized in Table 3. These plots result in equal correlation coefficients between 0.9800 and 0.9915 for the first peak of the As-cast state and the peaks of the HR and the HT conditions. In comparison, data obtained for the second peak of the As-cast condition show a lower correlation coefficient of 0.9327 which further leads to a larger scatter of the peak’s activation energy. It can be seen that the activation energies obtained for the first peak of the As-cast and the peaks of HR and HT conditions are between 34 ± 5 (HR and HT) and 38 ± 4 kJ/mol (As-cast). Due to the metrological uncertainty of ± 3 to 4 kJ/mol, the activation energies of these peaks do not differ significantly. For the second peak of the As-cast condition, an activation energy of 47 ± 8 kJ/mol was determined. Although the scattering ranges of the activation energies of both peaks deconvoluted from the As-cast state’s TDS spectrum are slightly overlapping, Figure 6a,b prove the presence of two clearly distinguishable peaks.

## 4. Discussion

### 4.1. Materials Characterization

The As-cast material solidifies from the melt. Its microstructure is dominated by coarse pearlite colonies oriented in the direction of solidification. Reheating the steel above its austenitization temperature, followed by hot rolling, leads to a significant change in its microstructure due to recrystallization processes. According to Table 1, the pearlite structure becomes finer when transitioning from the As-cast state to the HR and HT states. Microstructure and mechanical properties of pearlitic steels depend to a large amount on the cooling rate, which is indirectly proportional to the interlamellar spacing. The dependency of the mechanical properties on the interlamellar spacing is usually described using the Hall–Petch relationship so that a decrease in the interlamellar spacing results in an increase in yield strength, ultimate tensile strength, and hardness for non-oriented pearlite colonies [20,21,22,23]. This explains why the HT condition, which is head-hardened (quenched) and, therefore, provides the finest microstructure, shows the highest hardness values among the investigated conditions.

The oriented solidification structure of the As-cast state is modified during the heat treatment. The HR and HT conditions show the isotropic orientation of the pearlite colonies, as indicated by the IPF maps in Figure 3. Apart from the differences in orientation between the material conditions, EBSD data do not indicate any retained austenite, which could act as a hydrogen trap in any of the investigated materials. 

According to Calcagnotto et al. [24], KAM is directly related to the density of geometrical necessary dislocations (GND) following Equation (3).

(3) $$\rho_{GND}=\frac{2\cdot \vartheta }{u\cdot b}$$

where *ρ_GND_* gives the GND density, *ϑ* the misorientation angle, *u* the unit length, and *b* the magnitude of the Burgers vector. It has to be mentioned that KAM is based on GNDs and does not provide any information about statistically stored dislocations. Although this fact limits the derivation of the dislocation density, KAM can be considered a qualitative measure of the dislocation density [24,25,26,27]. As can be seen in the KAM map of the As-cast material (Figure 4b), the misorientation and, therefore, the dislocation density is lowest in big pearlite colonies with uniform orientation. Thus refinement of the pearlitic structure leads to an increase in dislocation density, as can be observed when comparing the KAM maps of the As-cast and the HT material states. The HT condition shows a more uniform distribution of zones with increased KAM values and an increased area where the misorientation angle is about 2°. When analyzing the HR condition, it has to be considered that after hot rolling, the cooling of the material is carried out on a cooling bed. While on this cooling bed, the material is cooled by the surrounding air. Therefore, a significantly lower cooling rate is applied compared with the HT condition. The lower cooling rate results in a significantly longer time for dislocations to heal and annihilate and, therefore, leads to a decrease in dislocation density compared to the HT state. The continuous increase in the dislocation density from the As-cast over the HR to the HT material state observed quantitatively by the comparison of the KAM factors was further confirmed using XRD measurements.

### 4.2. Electrochemical Permeation Measurement

Van den Eckhout et al. describe in [28] the influence of microstructural characteristics on the hydrogen permeation of steels. They conclude that trap density is detrimental to diffusion behavior because a high trap density leads to low diffusion coefficients and, therefore, to low diffusion rates. According to this, a high trap density results in longer lag times and shifts the increase in diffusion transient slopes towards longer charging times.

The effective hydrogen diffusion coefficients related to the As-cast and the HR material condition are both equal to 2.3 × 10^−6^ cm^2^/s and about four times higher than the effective hydrogen diffusion coefficient related to the HT (5.8 × 10^−7^ cm^2^/s) state. Due to the fact that dislocations act as hydrogen trapping sites in steel [29,30], the increasing dislocation density from the As-cast and HR to the HT condition was identified as a possible source for the difference in diffusion coefficients. The direct dependency of the effective hydrogen diffusion coefficient and the dislocation density has been well described in the literature [12,31,32,33]. Thereby, an increase in dislocation density leads to a decrease in the effective diffusion coefficient. Considering the breakthrough time of the As-cast and HR diffusion transients, a similar dislocation density can be assumed, which is also confirmed by the EBSD data and hardness measurements provided in Section 3.1. 

As described in [34], irreversible or so-called deep traps are filled up with hydrogen prior to reversible traps. Additionally, to the preferential uptake of hydrogen by deep traps, the high binding energies of these trapping sites are responsible for stronger hydrogen bonding. Therefore, hydrogen can hardly diffuse through the lattice at ambient temperature. Thus, apart from the trap density, the binding energy of the traps also influences the permeation transients in a way that a large concentration of irreversible traps, which are considered traps with an activation energy > 60 kJ/mol [28] and lead to an increase in the steepness of the permeation transients slope. 

When comparing the slopes of the diffusion transients (Figure 5a), it can be seen that the steepness of the HTs diffusion transient is significantly lower compared to the diffusion transients of the other material conditions. This leads, in combination with the increased breakthrough time, to the assumption that the HT material contains the highest trap density and the lowest ratio of deep to shallow traps. The slope of the As-cast material’s diffusion transient (Figure 5a) is slightly steeper than the one of HR, which indicates a higher ratio of deep to shallow traps in this material state. This assumption is supported by the results of the comparison between the first and second charging cycles. For the second charging cycle of the As-cast material, a shift in breakthrough time towards lower times is shown in Figure 4b. This indicates, as described in [35], that the material contains a significant amount of deep traps from which hydrogen cannot be removed during discharging. Therefore, these traps are already occupied with hydrogen prior to the second charging cycle, which leads to faster diffusion in the material and, thus, higher effective diffusion coefficients as well as lower breakthrough times. In the case of the HR and HT material conditions, this behavior was not observed, and diffusion transients, as well as effective diffusion coefficients, are not differing significantly for the first and second charging cycles. Because almost complete hydrogen effusion occurs during specimen discharging of HR and HT conditions, it can be assumed that trapping sites with lower activation energy predominate in both the HR and HT states compared with the As-cast state.

### 4.3. Thermal Desorption Spectroscopy

Analyzing the TDS spectra, a clear difference regarding the hydrogen trapping behavior of the tested material states can be distinguished. In principle, the investigated materials can be divided into two groups. The first group consists of the HR as well as the HT material condition, and the resulting sum curves of TDS spectra can be deconvoluted into a single peak. The As-cast state represents the second group, whereby its TDS spectrum can be deconvoluted into two peaks. Therefore, it can be assumed that the HR and the HT condition contain less active trapping sites than the As-cast material state. Table 4 provides a summary of literature data on the activation energies of trapping sites that can be present in the investigated material.

The calculation of the activation energy related to the first peak for all three states results in energies between 32 and 38 ± 4 kJ/mol. Considering the spectra given in Figure 6, it can be seen that the first peak of all material conditions has its maximum in a close temperature range between 60 and 70 °C (for an applied heating rate of 400 K/h). Due to this observation, it can be concluded that the hydrogen responsible for the first peak originates from the same trapping sites. It is unlikely that just one single active trap is present in HR and HT. It is reasonable to assume that different reversible traps with very similar activation energies are present next to each other, providing only one joint cumulative peak. Since the shape of the peak corresponds to the Gaussian normal distribution curve on which peak-fitting is based, it is not possible to further deconvolute this sum peak. Considering the calculated activation energy and the microstructural artifacts, dislocations, dislocation cores, grain boundaries, and the interface between ferrite and Fe_3_C are considered possible trapping sites contributing to the first peak. Activation energies for dislocations given in the literature are mostly between 20 and 34 kJ/mol [36,37,38,39,40,41]. Taketomi et al. show in their work [42] that the activation energy in an elastic stress field caused by a dislocation increases significantly with decreasing distance from the dislocation core. They report activation energies up to 42 kJ/mol directly at the dislocation core. To release hydrogen trapped at grain boundaries, an activation energy very similar to dislocations, which is specified to lie between 17 and 33 kJ/mol [19,37,43,44], is needed. For the activation energy of hydrogen trapped at the ferrite/Fe_3_C interface, it has to be clearly distinguished between the level of strain which was applied to the material because, in unstrained pearlitic steels, the ferrite/Fe_3_C interface shows activation energies between 23.5 and 31 kJ/mol [40,45,46]. However, when pearlitic material is deformed plastically, e.g., by cold drawing, an additional peak with activation energies between 90 and 105 kJ/mol occurs. This is related to hydrogen trapped at the strained ferrite/Fe_3_C interface [47,48]. When comparing the spectra of HR and HT, it can be seen that with an increasing amount of dislocations also, the peak height increases. This finding confirms the above-described contribution of dislocations to the cumulative peak obtained by TDS measurements.

**Figure 8:**
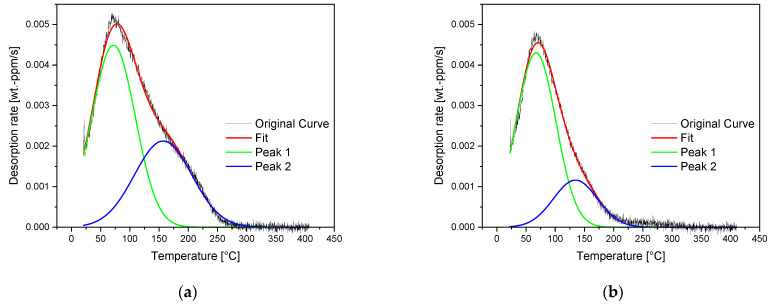
Deconvoluted TDS spectra were obtained from a heating rate of 400 K/h of As-cast bloom material at (**a**) 50% and (**b**) 75% of the peak height.

Identification of the second peak’s activation energy of the As-cast material results in 47 kJ/mol and is in good correlation with the value for microvoids, which was found to be 48.3 kJ/mol for low alloyed steel by Lee et al. [49]. In addition, the continuous casting process with its typical microvoids from the solidification process indicates a relation to microvoids. Near the center of the cast bloom section, there is a large number of microvoids. As indicated in Figure 3, the amount of microvoids decreases with increasing distance from the bloom’s center. Figure 8 compares the deconvoluted TDS spectra of specimens taken from as-cast bloom material at 50 (a) and 75% (b) of its height. It can be seen that the signal of the second peak is significantly more pronounced in the spectrum of the specimen taken at 50% of the bloom’s height compared with one of the specimens taken at 75% of its height. This is an indication that the amount of hydrogen in the trapping site regarding the second peak decreases over the bloom’s cross-section as the number of microvoids decreases. Microvoids are closed during hot-rolling, which can explain why a second peak can solely be observed in the As-cast condition. This is supported by the work of Laureys et al. [51], who showed by means of TDS analysis that microvoids can be annihilated by the application of annealing treatment. Therefore, the second peak of the As-cast material can be related to microvoids, responsible for the differences in diffusion coefficients of the first and second charging cycles described in the section above.

Non-metallic inclusions, especially MnS, are known to be initiation sites for hydrogen flaking [4,5,52]. In the present study, TDS spectra do not show evidence of hydrogen trapping directly at MnS, which leads to a hydrogen desorption peak with an activation energy of about 72 kJ/mol [49]. To act as an effective trapping site, inclusions must be small and finely dispersed; if MnS particles are relatively large in size, unevenly distributed, and in low density, the contribution to a materials desorption spectrum can be below the detection limit of TDS analysis.

## 5. Conclusions

The present study shows that hydrogen diffusion and trapping in pearlitic rail steel are not influenced to a significant amount by the material’s crystallographic orientation. Quenching of the material leads to a finer pearlite structure resulting in higher hardness. Furthermore, higher cooling rates result in a substantial increase in dislocation density confirmed using EBSD, XRD and TDS. The increase in dislocation density due to the applied heat treatment caused a decrease in the effective hydrogen diffusion coefficient by a factor of 4 and was determined to be around 5.8 × 10^−7^ cm^2^/s. According to data from electrochemical permeation and considering the TDS spectra, it can be assumed that hydrogen diffusion and trapping are controlled mainly by dislocation density in the finished rail materials (HR and HT). TDS analysis confirms the presence of a second peak related to an activation energy of 47.5 kJ/mol for the As-cast material that microvoids act as strong hydrogen traps. Although non-metallic inclusions, especially MnS, are discussed as possible initiation sides for hydrogen flaking in literature, TDS analysis did not show any evidence for MnS acting as a hydrogen trapping site in the investigated materials.

## Figures and Tables

**Table 1:** Summary of microstructural features present in the investigated material conditions.

Microstructural Feature	Material Condition		
	As-Cast	HR	HT
Pearlite colony size	coarse	fine	very fine
Texture	dendritic oriented	none	none
Hardness [HV]	278 ± 3	285 ± 7	364 ± 8
Microvoids	yes	no	no
KAM	0.62	0.81	1.10
Dislocation density [m^−2^]	2.1 × 10^14^	3.9 × 10^14^	9.3 × 10^14^
Lattice structure	bcc	bcc	bcc
LAGB [%]	88.2	78.5	80.3
HAGBs [%]	11.8	21.5	19.7

**Table 2:** Diffusion coefficients of the investigated materials.

Material Condition	*D_eff_*_1_ [cm²/s]	*D_eff_*_2_ [cm²/s]
As-cast	2.3 × 10^−6^	4.3 × 10^−6^
HR	2.3 × 10^−6^	3.0 × 10^−6^
HT	5.8 × 10^−7^	5.8 × 10^−7^

**Table 3:** Summary of the trap activation energies obtained by TDS measurements.

Material Condition	Activation Energy Peak 1 (*E_A_*_1_) [kJ/mol]	Activation Energy Peak 2 (*E_A_*_2_) [kJ/mol]
As-cast	38 ± 4	47 ± 8
HR	34 ± 4	
HT	32 ± 3	

**Table 4:** Summary of literature data on activation energies of hydrogen traps.

Trapping Side	Material	E_A_ [kJ/mol]	Reference
Dislocations	Pure iron, Low alloyed steel	20–34	[36,37,38,39,40,41]
Dislocation cores	Pure iron	~42	[42]
Grain boundaries	Pure iron, Low alloyed steel	17–33	[19,37,43,44]
Fe/Fe_3_C interface unstrained	Pearlite, Low alloyed steel	~24–31	[40,45,46]
Fe/Fe_3_C interface strained	Pearlite	90–105	[47,48]
Microvoids	Pure iron, Low alloyed steel	40–50	[49,50]
MnS	Low alloyed steel	~72	[49]

## Data Availability

The data presented in this study are available on request from the corresponding author.

## References

[1] Dean S.W., Sahay S.S., Mohapatra G., Totten G.E. (2009). Overview of Pearlitic Rail Steel: Accelerated Cooling, Quenching, Microstructure, and Mechanical Properties. J. ASTM Int..

[2] Kuziak R., Zygmunt T. (2012). A New Method of Rail Head Hardening of Standard-Gauge Rails for Improved Wear and Damage Resistance. Steel Res. Int..

[3] Panda B., Balasubramaniam R., Moon A. (2009). Microstructure and mechanical properties of novel rail steels. Mater. Sci. Technol..

[4] Laureys A., Van Stappen J., Depover T., Cnudde V., Verbeken K. (2019). Electrochemical hydrogen charging to simulate hydrogen flaking in pressure vessel steels. Eng. Fract. Mech..

[5] Ravichandar D., Balusamy T., Balachandran G. (2019). Behaviour of Hydrogen During the Manufacture of Rail Steels. Trans. Indian Inst. Met..

[6] Uyama H., Yamada H., Hidaka H., Mitamura N. (2011). The Effects of Hydrogen on Microstructural Change and Surface Originated Flaking in Rolling Contact Fatigue. Tribol. Online.

[7] Gao N., Wei-Xun Y., Yin-Zhi C. (1992). Flakes in low carbon high strength low alloy steel. Mater. Charact..

[8] Voronenko B.I. (1997). Hydrogen and flakes in steel. Met. Sci. Heat Treat..

[9] Ravichandar D., Balusamy T., Gobinath R., Balachandran G. (2018). Behaviour of Hydrogen in Industrial Scale Steel Melts. Trans. Indian Inst. Met..

[10] Pillot S., Coudreuse L., Gangloff R.P., Somerday B.P. (2012). Hydrogen-Induced Disbonding and Embrittlement of Steels Used in Petrochemical Refining. Gaseous Hydrogen Embrittlement of Materials in Energy Technologies.

[11] Fan J., Chen H., Zhao W., Yan L. (2018). Study on Flake Formation Behavior and Its Influence Factors in Cr5 Steel. Materials.

[12] Drexler A., Siegl W., Ecker W., Tkadletz M., Klösch G., Schnideritsch H., Mori G., Svoboda J., Fischer F. (2020). Cycled hydrogen permeation through Armco iron—A joint experimental and modeling approach. Corros. Sci..

[13] Wilkens M. (1970). The determination of density and distribution of dislocations in deformed single crystals from broadened X-ray diffraction profiles. Phys. Status Solidi.

[14] Krivoglaz M.A. (1984). Influence of correlation in position of dislocations on x-ray diffraction by deformed crystals. Phys. Met. Met..

[15] Krivoglaz M.A., Ryaboshapka K.P. (1963). Theory of X-ray scattering by crystals containing dislocations, screw and edge disloca-tions randomly distributed throughout the crystal. Fiz. Met. Met..

[16] Devanathan M.A.V., Stachurski Z. (1962). The adsorption and diffusion of electrolytic hydrogen in palladium. Proc. R. Soc. Lond. Ser. A Math. Phys. Sci..

[17] Austrian Standards Institute (2014). Elektrochemisches Verfahren zur Messung der Wasserstoffpermeation und zur Bestimmung von Wasserstoffaufnahme und -Transport in Metallen.

[18] Kissinger H.E. (1957). Reaction Kinetics in Differential Thermal Analysis. Anal. Chem..

[19] Choo W.Y., Lee J.Y. (1982). Thermal analysis of trapped hydrogen in pure iron. Met. Trans. A.

[20] Toribio J., González B., Matos J.-C., Ayaso F.-J. (2016). Influence of Microstructure on Strength and Ductility in Fully Pearlitic Steels. Metals.

[21] Modi O., Desmukh N., Mondal D., Jha A., Yegneswaran A., Khaira H. (2001). Effect of interlamellar spacing on the mechanical properties of 0.65% C steel. Mater. Charact..

[22] Marder A.R., Bramfitt B.L. (1976). The effect of morphology on the strength of pearlite. Met. Trans. A.

[23] Elwazri A., Wanjara P., Yue S. (2005). The effect of microstructural characteristics of pearlite on the mechanical properties of hypereutectoid steel. Mater. Sci. Eng. A.

[24] Calcagnotto M., Ponge D., Demir E., Raabe D. (2010). Orientation gradients and geometrically necessary dislocations in ultrafine grained dual-phase steels studied by 2D and 3D EBSD. Mater. Sci. Eng. A.

[25] Guglielmi P.O., Ziehmer M., Lilleodden E.T. (2018). On a novel strain indicator based on uncorrelated misorientation angles for correlating dislocation density to local strength. Acta Mater..

[26] Jiang J., Britton T., Wilkinson A. (2013). Evolution of dislocation density distributions in copper during tensile deformation. Acta Mater..

[27] Li H., Hsu E., Szpunar J., Utsunomiya H., Sakai T. (2008). Deformation mechanism and texture and microstructure evolution during high-speed rolling of AZ31B Mg sheets. J. Mater. Sci..

[28] Eeckhout E.V.D., Depover T., Verbeken K. (2018). The Effect of Microstructural Characteristics on the Hydrogen Permeation Transient in Quenched and Tempered Martensitic Alloys. Metals.

[29] Choo W.Y., Lee J.Y. (1982). Hydrogen trapping phenomena in carbon steel. J. Mater. Sci..

[30] Shi R., Chen L., Wang Z., Yang X.-S., Qiao L., Pang X. (2021). Quantitative investigation on deep hydrogen trapping in tempered martensitic steel. J. Alloys Compd..

[31] Luppo M., Ovejero-Garcia J. (1991). The influence of microstructure on the trapping and diffusion of hydrogen in a low carbon steel. Corros. Sci..

[32] Eeckhout E.V.D., Laureys A., Van Ingelgem Y., Verbeken K. (2017). Hydrogen permeation through deformed and heat-treated Armco pure iron. Mater. Sci. Technol..

[33] Siegl W., Ecker W., Klarner J., Kloesch G., Mori G., Drexler A., Winter G., Schnideritsch H. Hydrogen trapping in heat treated and deformed Armco iron. Proceedings of the CORROSION 2019.

[34] Dadfarnia M., Sofronis P., Neeraj T. (2011). Hydrogen interaction with multiple traps: Can it be used to mitigate embrittlement?. Int. J. Hydrogen Energy.

[35] Chen L., Antonov S., Su Y., Qiao L. (2022). Dislocation cell walls with high dislocation density as effective hydrogen traps in Armco iron. Mater. Corros..

[36] Hirth J.P. (1980). Effects of hydrogen on the properties of iron and steel. Met. Trans. A.

[37] Pressouyre G.M. (1979). A classification of hydrogen traps in steel. Met. Trans. A.

[38] Takai K., Chiba Y., Noguchi K., Nozue A. (2002). Visualization of the hydrogen desorption process from ferrite, pearlite, and graphite by secondary ion mass spectrometry. Met. Mater. Trans. A.

[39] Wei F.-G., Tsuzaki K. (2005). Response of hydrogen trapping capability to microstructural change in tempered Fe–0.2C martensite. Scr. Mater..

[40] Pinson M., Claeys L., Springer H., Bliznuk V., Depover T., Verbeken K. (2022). Investigation of the effect of carbon on the reversible hydrogen trapping behavior in lab-cast martensitic Fe C steels. Mater. Charact..

[41] Moshtaghi M., Loder B., Safyari M., Willidal T., Hojo T., Mori G. (2022). Hydrogen trapping and desorption affected by ferrite grain boundary types in shielded metal and flux-cored arc weldments with Ni addition. Int. J. Hydrogen Energy.

[42] Taketomi S., Matsumoto R., Miyazaki N. (2008). Atomistic study of hydrogen distribution and diffusion around a {112}<111> edge dislocation in alpha iron. Acta Mater..

[43] Wallaert E., Depover T., Arafin M., Verbeken K. (2014). Thermal Desorption Spectroscopy Evaluation of the Hydrogen-Trapping Capacity of NbC and NbN Precipitates. Met. Mater. Trans. A.

[44] Vandewalle L., Konstantinović M.J., Verbeken K., Depover T. (2022). A combined thermal desorption spectroscopy and internal friction study on the interaction of hydrogen with microstructural defects and the influence of carbon distribution. Acta Mater..

[45] Hagi H. (1994). Effect of Interface between Cementite and Ferrite on Diffusion of Hydrogen in Carbon Steels. Mater. Trans. JIM.

[46] Truschner M., Pengg J., Loder B., Köberl H., Gruber P., Moshtaghi M., Mori G. (2023). Hydrogen resistance and trapping behaviour of a cold-drawn ferritic–pearlitic steel wire. Int. J. Mater. Res..

[47] Yu S.-H., Lee S.-M., Lee S., Nam J.-H., Lee J.-S., Bae C.-M., Lee Y.-K. (2019). Effects of lamellar structure on tensile properties and resistance to hydrogen embrittlement of pearlitic steel. Acta Mater..

[48] Doshida T., Takai K. (2014). Dependence of hydrogen-induced lattice defects and hydrogen embrittlement of cold-drawn pearlitic steels on hydrogen trap state, temperature, strain rate and hydrogen content. Acta Mater..

[49] Lee J.L., Lee J.Y. (1983). Hydrogen trapping in AISI 4340 steel. Met. Sci..

[50] Lee J.-Y., Lee J.-L. (1987). A trapping theory of hydrogen in pure iron. Philos. Mag. A.

[51] Laureys A., Claeys L., Pinson M., Depover T., Verbeken K. (2020). Thermal desorption spectroscopy evaluation of hydrogen-induced damage and deformation-induced defects. Mater. Sci. Technol..

[52] De Bruycker E., De Vroey S., Huysmans S., Stubbe J. (2014). Phenomenology of Hydrogen Flaking in Nuclear Reactor Pressure Vessels. Mater. Test..

